# Takotsubo cardiomyopathy secondary to alcohol withdrawal

**DOI:** 10.21542/gcsp.2022.6

**Published:** 2022-06-30

**Authors:** Faizan Ahmed, Jeffrey F. Spindel, Agastya Belur, Shahab Ghafghazi

**Affiliations:** 1 University of Louisville School of Medicine, Department of Internal Medicine; 2 University of Louisville School of Medicine, Division of Cardiovascular Medicine

## Abstract

Physical, mental, and emotional stressors have been well known to adversely affect cardiac function. A rare complication of such stressors is stress cardiomyopathy, otherwise known as takotsubo cardiomyopathy. First identified in Japan in the 1990s, takotsubo cardiomyopathy classically presents with systolic dysfunction and apical ballooning. In this report, we present the case of a patient with a medical history of alcohol abuse who presented to the emergency department after being found unresponsive by her family. Transthoracic echocardiography revealed takotsubo cardiomyopathy, which was likely secondary to alcohol withdrawal. Alcohol withdrawal causes an imbalance between various neurotransmitters such as GABA and glutamate. This imbalance caused autonomic overactivity, which manifested as stress cardiomyopathy.

## Introduction

Takotsubo cardiomyopathy, a non-ischemic cardiomyopathy characterized by transient systolic dysfunction with apical ballooning, often mimics the presentation of an acute coronary syndrome (ACS) due to the presence of left ventricular regional wall motion abnormality and troponin elevation^1^. In 1990, researchers in Japan reported this reversible form of cardiomyopathy, which presented with an apical ballooning pattern and akinesis despite the lack of obstructive coronary artery disease^2^.

Classically, this form of cardiomyopathy presents after a significant emotional or physical stressor, more commonly in women than in men. The pathophysiology is incompletely understood, but is likely multifactorial, consisting of multivessel spasms, coronary microvascular impairment, and catecholamine surge.

Although it is associated with emotional or physical stressors, alcohol abuse has also been implicated in the development of stress cardiomyopathy. Chronic exposure alters the neurochemistry of the brain by altering the balance between the excitatory and inhibitory transmitters. Sudden withdrawal from alcohol causes a surge of catecholamines which is suspected to play a primary role in the development of takotsubo cardiomyopathy due to alcohol withdrawal.

### Case presentation

A 47-year-old female with a medical history of alcohol use disorder, ulcerative colitis, hypertension, and depression presented to the Emergency Department after being found unresponsive by her family. The family reported that she drank six shots of liquor per day, with her last drink being two days prior to admission. Emergency medical services witnessed two seizures with the longest lasting 2 min and intubated the patient for airway protection. The patient was afebrile, tachypneic with a respiratory rate of 22 breaths/min, and hypotensive with a blood pressure of 96/54 mmHg.

Initial lab values were significant for lactate of 4.5 mmol/L, glucose of 231 mg/dl, creatinine of 1.27 mg/dl, white blood cell count of 12.9 × 10^3^/µL, AST of 157 units/liter, ALT of 52 units/liter, and troponin I of 0.58 ng/mL. Initial EKG showed ST depressions in the inferior leads and sinus tachycardia with a rate of 133 beats per minute. The patient was admitted to the intensive care unit.

She was treated with aspirin, atorvastatin, and a heparin drip for non-ST-elevation myocardial infarction (NSTEMI) with a troponin peak of 2.20 mg/dL and required inotropic support for undifferentiated shock.

Transthoracic echocardiogram (TTE) with contrast revealed severely reduced left ventricular ejection fraction with apical and mid-segmental akinesis but normal contraction of basal segments, a pattern consistent with takotsubo cardiomyopathy.

**Figure 1.**
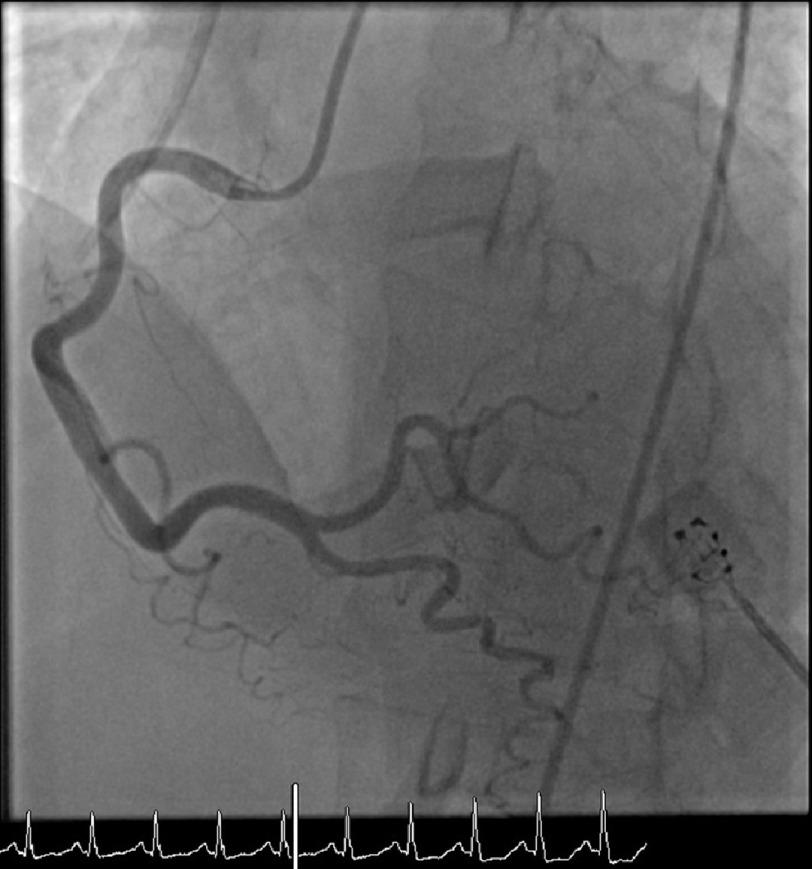
RAO caudal views revealing no significant coronary artery disease of the left-sided coronary arteries.

**Figure 2.**
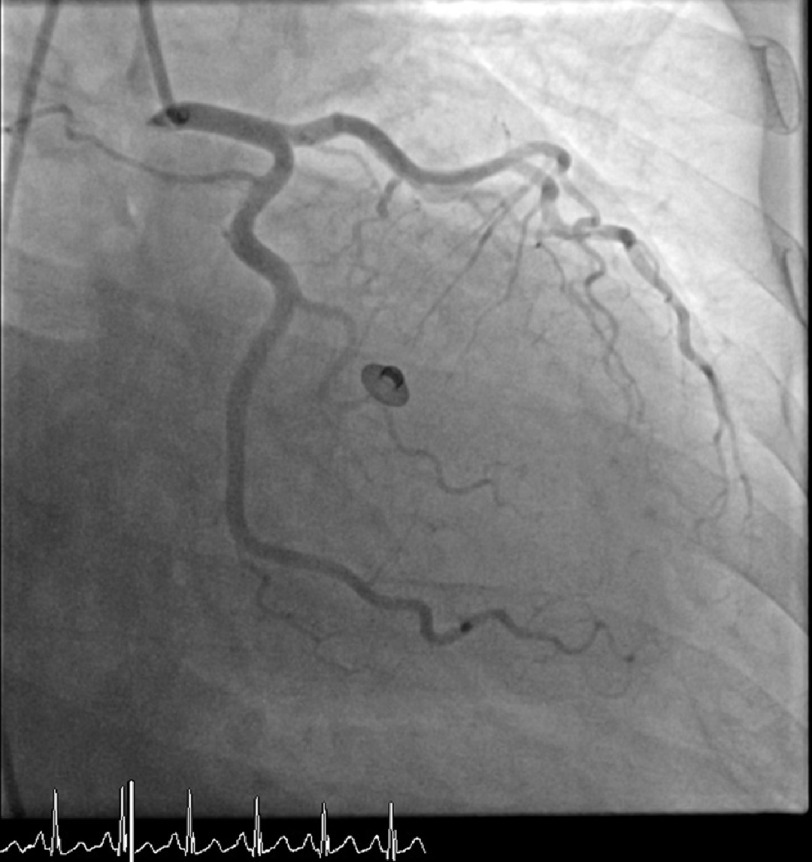
LAO cranial view revealing no significant coronary artery disease of the right-sided coronary arteries.

Right heart catheterization revealed a right atrial mean pressure of 0 mmHg, right ventricular pressure of 17/0 mmHg, pulmonary artery pressure of 13/5 mmHg, and PCW pressure of 3 mmHg, indicating intravascular volume depletion. Coronary angiography demonstrated a left ventricular end-diastolic pressure of 32 mmHg and non-obstructive coronary artery disease. [Figure 1 and 2] With gentle fluid resuscitation the patient’s blood pressure improved. With supportive care, she gradually stabilized, and her lactic acid and troponin levels trended to normal. After extubation, the patient showed clear mentation and an interest in resources to support alcohol cessation.

During hospitalization, the patient was started on levetiracetam for seizure prophylaxis and required four days of benzodiazepines for alcohol withdrawal. No further seizures occurred, the EEG was negative for ictal tendencies, and lumbar puncture revealed no signs of central nervous system infection. The patient was discharged after an 8-day hospitalization.

## Discussion

Takotsubo or stress cardiomyopathy can mimic ACS with ST changes and troponin elevation. Therefore, it is a diagnosis of exclusion in addition to support from characteristic echocardiographic findings of transient hypokinesis, akinesis, or dyskinesis of the left ventricular apex with or without involvement of middle left ventricular segments and regional wall motion abnormalities that extend beyond a single coronary artery’s epicardial distribution^3^.

Stress cardiomyopathy is usually elicited by physical or emotional stressors, such as critical medical illness, and is thought to be due catecholaminergic overload^4^. Overstimulation of cardiac myocytes through beta-adrenergic agonism increases heart rate and contractility leading to an imbalance in oxygen supply and demand. Arterial vasospasm and vasoconstriction from alpha-adrenergenic stimulation further results in myocardial ischemia and cell death, leading to characteristic ST changes on ECG and troponin elevation^5^. Overstimulation of the basal portion of the heart can cause left ventricular outflow tract obstruction and hypotension, which in turn requires further vasopressor support and can further complicate medical management. In support of this hypothesis, plasma levels of both epinephrine and norepinephrine were remarkably elevated in patients with stress cardiomyopathy^6^.

While normally attributed to mental stress, trauma, or surgery, this patient suffered stress cardiomyopathy due to alcohol withdrawal. Chronic alcohol ingestion affects various neurotransmitters such as GABA, an inhibitory neurotransmitter, and glutamate, an excitatory neurotransmitter. Due to the unbalanced neurochemistry, abrupt cessation of alcohol leads to autonomic overactivity, frequently causing tachycardia, tremors, diaphoresis, and if untreated, seizures^7^. Here, we present a catecholaminergic surge from alcohol withdrawal as the cause of stress cardiomyopathy.

### What have we learned?

• Stress cardiomyopathy is similar to acute coronary syndrome, and typically occurs after significant physical or emotional stressors.
• Stress cardiomyopathy is a diagnosis of exclusion, though the classic echocardiographic findings are transient ballooning of the left ventricular apex and regional wall motion abnormalities extending beyond a single coronary artery’s epicardial distribution.
• Alcohol withdrawal can induce cardiomyopathy due to an imbalance between neurotransmitters and catecholamines.

### Authors’ statement

**Conceptualization:** Jeffrey F. Spindel. **Data curation:** Agastya Belur. **Formal analysis:** Jeffrey F. Spindel and Agastya Belur. **Investigation:** Faizan Ahmed. **Supervision:** Shahab Ghafghazi. **Validation:** Shahab Ghafghazi. **Writing - original draft:** Faizan Ahmed. **Writing - review & editing:** Faizan Ahmed, Jeffrey F. Spindel, Agastya Belur, and Shahab Ghafghazi.

### Editor’s note

There are 3 videos available as supplementary files in the online version of this article.
